# Supplementary material for: Dynamic omnivory shapes the functional role of large carnivores under global change
Source: Nat Commun. 2025 Dec 3;16:10896. doi: 10.1038/s41467-025-65959-7 (PMC12678555; doi:10.1038/s41467-025-65959-7)
Supplement: Supplementary file 1 — Supplementary Information [file 41467_2025_65959_MOESM1_ESM.pdf]

Supplementary Information for

Dynamic omnivory shapes the functional role of large carnivores under global change

Authors

Jörg Albrecht[1,2], Hervé Bocherens[3,4], Keith A. Hobson[5,22], Dorothée G. Drucker[3], Agnieszka Sergiel[2], Jon E. Swenson[6], Andreas Zedrosser[7,8], Adrian Marciszak[9], Elisabeth Iregren[10], Leena Drenzel[11], René Kyselý[12], Grzegorz Lipecki[13], Daniel Makowiecki[14], Jan Wagner[15], Tomasz Zwijacz-Kozica[16], Susanne A. Fritz[1,17,18,19], Eloy Revilla[20] & Nuria Selva[2,20,21]

Affiliations

[1]Senckenberg Biodiversity and Climate Research Centre (SBiK-F), Senckenberganlage 25, Frankfurt am Main 60325, Germany.

[2]Institute of Nature Conservation, Polish Academy of Sciences, Mickiewicza 33, PL-31-120, Kraków, Poland.

[3]Senckenberg Centre for Human Evolution and Palaeoenvironment (SHEP), University of Tübingen, Tübingen, Germany.

[4]Department of Geosciences (Biogeology), University of Tübingen, Tübingen, Germany.

[5]Environment and Climate Change Canada, Canada. Dept Biology, University of Western Ontario, London, Canada.

[6]Faculty of Environmental Sciences and Natural Resource Management, Norwegian University of Life Sciences, Ås, Norway.

[7]Department of Natural Sciences and Environmental Health, University of South-Eastern Norway, Bø, Norway.

[8]Institute for Wildlife Biology and Game Management, University for Natural Resources and Life Sciences, Vienna, Gregor Mendel Str. 33, A-1180 Vienna, Austria.

[9]Department of Paleozoology, Faculty of Biological Sciences, University of Wrocław, Wrocław, Poland.

[10]Department of Archaeology and Ancient History, Lund University, Sweden.

[11]Department of Cultural History and Collections, National Historical Museum, P.O.Box 5428, SE-114 84 Stockholm, Sweden.

[12]Department of Natural Sciences and Archaeometry, Institute of Archaeology of the Czech Academy of Sciences, Prague, Czech Republic.

[13]Institute of Systematics and Evolution of Animals, Polish Academy of Sciences, Sławkowska 17, 31-016, Kraków, Poland.

[14]Department of Environmental Archaeology and Human Paleoecology, Institute of Archaeology, Nicolaus Copernicus University of Toruń, Toruń, Poland.

[15]Department of Palaeontology, National Museum, Prague, Czech Republic.

[16]Tatra National Park, 34-500 Zakopane, Poland.

[17]Goethe University Frankfurt, Institut für Geowissenschaften, Altenhöferallee 1, 60438 Frankfurt am Main.

[18]German Centre for integrative Biodiversity Research (iDiv) Halle-Jena-Leipzig, Puschstr. 4, 04103 Leipzig.

[19]Friedrich Schiller University Jena, Institute of Biodiversity, Ecology and Evolution, Dornburger Str. 159, 07743 Jena.

[20]Estacion Biológica de Doñana Consejo Superior de Investigaciones Científicas (CSIC), 41092 Sevilla, Spain.

[21]Departamento de Ciencias Integradas, Facultad de Ciencias Experimentales, Centro de Estudios Avanzados en Física, Matemáticas y Computación, Universidad de Huelva, 21071 Huelva, Spain.

[22]Deceased: Keith A. Hobson.

Supplementary Table 1. Summary of dietary classification scheme and associated correction factors.

$diet_1$	$diet_2$	$diet_3$	c_D	c_E	comment	c_D reference	c_E reference
animals	vertebrates	endotherm_vertebrates	3.125	14.200	–	1–4	2
animals	vertebrates	ectotherm_vertebrates	3.125	14.200	–	1–4	2
animals	vertebrates	fish_vertebrates	40.800	14.200	–	–	–
animals	vertebrates	unknown_vertebrates	3.125	14.200	mean of endo and ecto vertebrates	–	–
animals	invertebrates	aquatic_invertebrates	1.100	14.500	assumed same as terrestrial invertebrates	1,3,4	3,4
animals	invertebrates	terrestrial_invertebrates	1.100	14.500	–	1,3,4	3,4
animals	invertebrates	unknown_invertebrates	1.100	14.500	mean of aquatic and terrestrial invertebrates	–	–
animals	unknown_animal_material	unknown_animal_material	2.113	14.350	mean of vertebrates and invertebrates	–	–
plants	reproductive_plant_material	fruit	1.064	18.100	–	1,5	6
plants	reproductive_plant_material	seeds	1.450	26.700	–	1,5	6
plants	reproductive_plant_material	flowers_nectar_pollen	0.270	8.400	assumed same as forbs, herbs, legumes	1	3,4
plants	reproductive_plant_material	unknown_reproductive_plant_material	0.928	17.733	mean of reproductive plant materials	–	–
plants	vegetative_plant_material	forbs_herbs_legumes	0.270	8.400	–	1	3,4
plants	vegetative_plant_material	grass	0.230	6.300	–	1	3,4
plants	vegetative_plant_material	roots_tubers_bulbs	0.800	16.500	–	1	6
plants	vegetative_plant_material	leaves_branches_bark	0.270	8.400	assumed same as forbs, herbs, legumes	1	3,4
plants	vegetative_plant_material	unknown_vegetative_plant_material	0.393	9.900	mean of vegetative plant materials	–	–
plants	unknown_plant_material	unknown_plant_material	0.660	13.817	mean of reproductive and vegetative plant materials	–	–
fungi_lichens_bryophytes_algae	fungi_lichens_bryophytes_algae	fungi_lichens_bryophytes_algae	0.270	8.400	assumed same as forbs, herbs, legumes	1	3,4
other	other	other	0.000	0.000	–	–	–

Note: $Diet_1$, $Diet_2$, and $Diet_3$ are dietary classifications at different resolutions. Provided are also correction factors (and literature sources) that account for differences in digestibility (c_D) and energy content (c_E) of food items in $Diet_3$.

Supplementary Table 2. Summary of Bayesian hierarchical model testing for relationships of the trophic position (i.e., relative dietary energy contribution of animal prey) with net primary productivity (NPP), growing season length (GSL), and competition with sympatric bear species across the geographic ranges of the seven extant terrestrial bear species.

Source of variance	Median	5%	95%	p_d	VIF	PSRF	N_{eff}
(**a**) Sub-model I ($V \sim F$), PPP = 0.4947							
Intercept	0.427	0.287	0.563	1	–	1	10000
Slope	1.31	1.23	1.4	1	–	1	10000
$\sigma^2_{intercept}$	0.787	0.635	0.994	–	–	1	10000
σ^2_{slope}	0.283	0.229	0.356	–	–	1	10000
$\rho_{intercept,slope}$	0.955	0.938	0.967	–	–	1	10000
$\sigma^2_{residual}$	0.723	0.665	0.786	–	–	1	10000
r^2	0.828	0.809	0.847	–	–	1	9406
(**b**) Sub-model II (Intercept only), PPP = 0.5086							
Intercept	-0.783	-1.15	-0.462	0.999	–	1	10000
$\sigma^2_{species}$	0.152	0.0402	0.607	–	–	1.01	9528
σ^2_{study}	0.519	0.401	0.672	–	–	1	10000
$\sigma^2_{residual}$	0.133	0.0966	0.187	–	–	1	10000
$r^2_{marginal}$	3.73E-30	2.03E-32	2.22E-29	–	–	1	0
$r^2_{conditional}$	0.637	0.393	0.778	–	–	1	9693
(**c**) Sub-model II (NPP + GSL + competition), PPP = 0.5041							
Intercept	-0.547	-0.933	-0.159	0.982	–	1	11111
Log_{10} Net primary productivity (Log_{10} NPP)	-0.183	-0.303	-0.0655	0.994	1.52	1	9658
Growing season length (GSL)	-0.414	-0.569	-0.264	1	1.97	1	10000
Competition (subordinate)	-0.504	-0.843	-0.158	0.991	1.37	1	10000
Competition (dominant)	0.0586	-0.258	0.366	0.616	1.08	1	10000
$\sigma^2_{species}$	0.206	0.0213	0.98	–	–	1.01	10000
σ^2_{study}	0.318	0.223	0.432	–	–	1	10000
$\sigma^2_{residual}$	0.136	0.0992	0.196	–	–	1	10000
$r^2_{marginal}$	0.276	0.149	0.394	–	–	1	10000
$r^2_{conditional}$	0.625	0.34	0.801	–	–	1	10000
(**d**) Sub-model II (Two-component model), PPP = 0.5010							
Intercept	-0.744	-1.41	-0.261	0.986	–	1	10000
$Log_{10}\ NPP_{ij}$ – mean($Log_{10}\ NPP_{ij})_j$	-0.178	-0.3	-0.0577	0.992	1.43	1	10000
mean($Log_{10}\ NPP_{ij})_j$	-0.229	-1.05	0.726	0.683	1.4	1	10000
GSL_{ij} – mean($GSL_{ij})_j$	-0.44	-0.596	-0.284	1	1.94	1	10798
mean($GSL_{ij})_j$	0.0561	-0.756	1.06	0.553	1.4	1	10000
Competition (subordinate)	-0.52	-0.862	-0.175	0.993	1.43	1	10000
Competition (dominant)	0.0678	-0.245	0.389	0.644	1.09	1	10000
$\sigma^2_{species}$	0.282	0.0394	1.39	–	–	1.02	9712
σ^2_{study}	0.312	0.219	0.42	–	–	1	10000
$\sigma^2_{residual}$	0.137	0.0998	0.195	–	–	1	10068
$r^2_{marginal}$	0.25	0.129	0.397	–	–	1	9602
$r^2_{conditional}$	0.573	0.315	0.769	–	–	1	9590

Given are estimated model parameters [median and 90% Equal-Tailed Intervals (ETIs)], and convergence diagnostics [potential scale reduction factor (PSRF) and effective sample size (N_{eff})] for all model parameters. The fraction of posterior samples with the same sign as the median (p_d) is provided as a measure of support for the effects of the explanatory variables. Variance inflation factors (VIFs) quantify the variance inflation of effect sizes due to collinearity among predictor variables, with VIF > 10 indicating strong collinearity[7]. PPP-values for each sub-model indicate how well model simulations fit the observed data. Values of PPP close to 0.5 indicate that the model fits the observed data, while values close to 0 or 1 indicate the opposite.

Supplementary Table 3. Summary of Bayesian hierarchical model testing for relationships of trophic position (i.e., trophic level) with net primary productivity (NPP) and growing season length (GSL) in the brown bear (*Ursus arctos*) from the Late Pleistocene to the Holocene.

Source of variance	Median	5%	95%	p_d	VIF	PSRF	N_{eff}
(**a**) Sub-model I							
Effect of elevation, PPP = 0.5069							
Tissue type							
Vegetation	2.87	2.47	3.28	1	–	1	9835
Sheep wool	6.46	5.97	6.94	1	–	1	10837
Goat hair	5.94	5.39	6.47	1	–	1	10000
Cattle hair	6.09	5.31	6.86	1	–	1	10000
Elevation ($\beta_{Elevation}$)	-0.0013	-0.00154	-0.00106	1	–	1	10000
$\sigma^2_{residual}$	1.14	0.867	1.55	–	–	1	10000
Effect of sampled material (bone vs. tooth), PPP = 0.4893							
μ_T	1.51	1.31	1.7	1	–	1	10000
σ^2_T	0.544	0.373	0.842	–	–	1	10000
Trophic discrimination factor, PPP = 0.5060							
μ_Δ	3.9	3.34	4.54	1	–	1	10000
σ^2_Δ	0.845	0.358	1.67	–	–	1	10000
(**b**) Sub-model II, PPP = 0.4157							
Intercept	2.34	2.22	2.5	1	–	1	10000
Log_{10} Net primary productivity (Log_{10} NPP)	-0.173	-0.304	-0.0292	0.97	1	1	10000
Growing season length (GSL)	-0.185	-0.325	-0.0411	0.976	1	1	9643
$\sigma^2_{residual}$	0.0192	0.000427	0.141	–	–	1.02	10000
r^2	0.776	0.217	0.994	–	–	1	10000

Given are estimated model parameters [median and 90% Equal-Tailed Intervals (ETIs)], and convergence diagnostics [potential scale reduction factor (PSRF) and effective sample size (N_{eff})] for all model parameters. The fraction of posterior samples with the same sign as the median (p_d) is provided as a measure of support for the effects of the explanatory variables. Variance inflation factors (VIFs) quantify the variance inflation of effect sizes due to collinearity among predictor variables, with VIF > 10 indicating strong collinearity[7]. PPP-values for each sub-model indicate how well model simulations fit the observed data. Values of PPP close to 0.5 indicate that the model fits the observed data, while values close to 0 or 1 indicate the opposite.

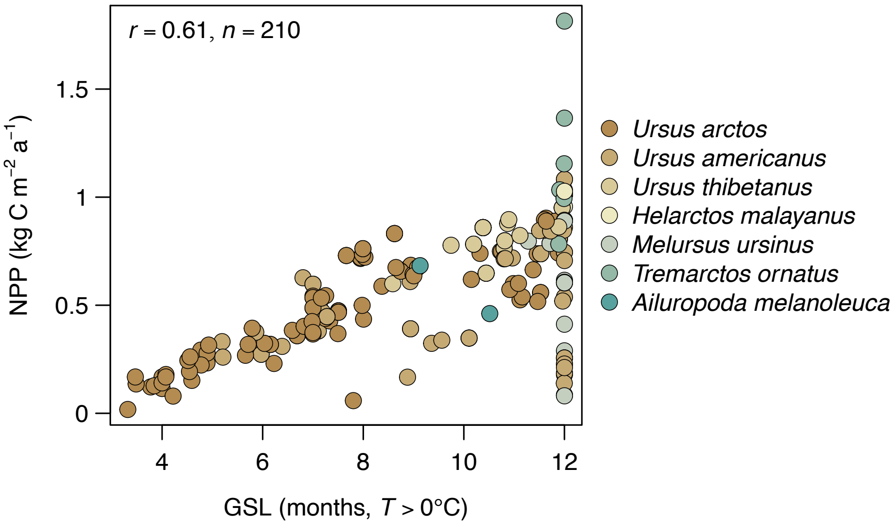

Supplementary Figure 1. Relationship between growing season length (GSL, months $T > 0$°C) and net primary productivity (NPP, kg C m^{-2} a^{-1}) across study locations in the macroecological data. NPP and GSL were moderately positively correlated (Pearson's r = 0.61, $t = 11.2$, df = 208, $P < 0.001$). Sample size is indicated by n.

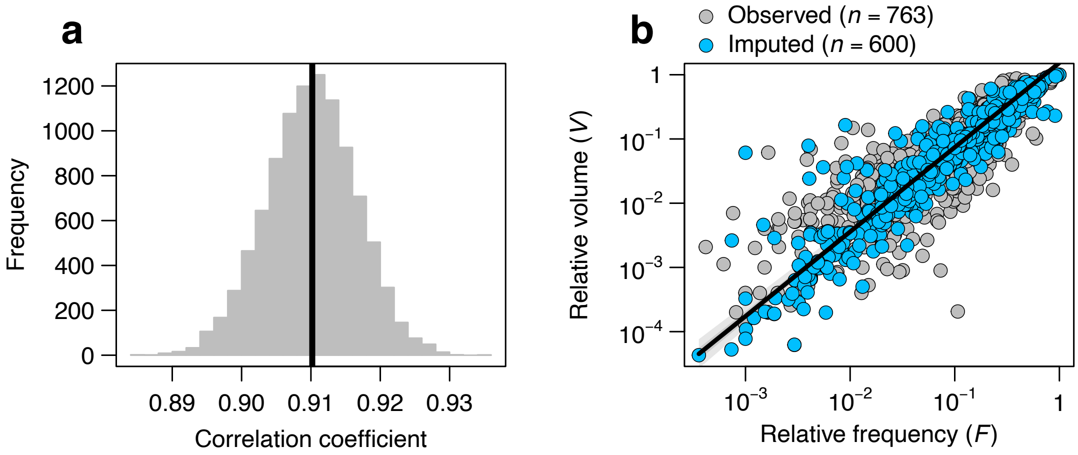

Supplementary Figure 2. Relationship between relative frequency (*F*) and relative volume (*V*) of dietary food items. a, Median (black line) and posterior distribution of estimated correlation coefficient between *F* and *V* based on sub-model I in the macroecological analysis (Supplementary Table 2a). **b**, Geometric mean regression relationship between *F* and *V* for observed (white dots) and imputed (blue dots) data. Black line, dark and light gray bands represent the estimated relationship and uncertainty (median, 50% and 90% ETIs, respectively). Sample size is indicated by *n*.

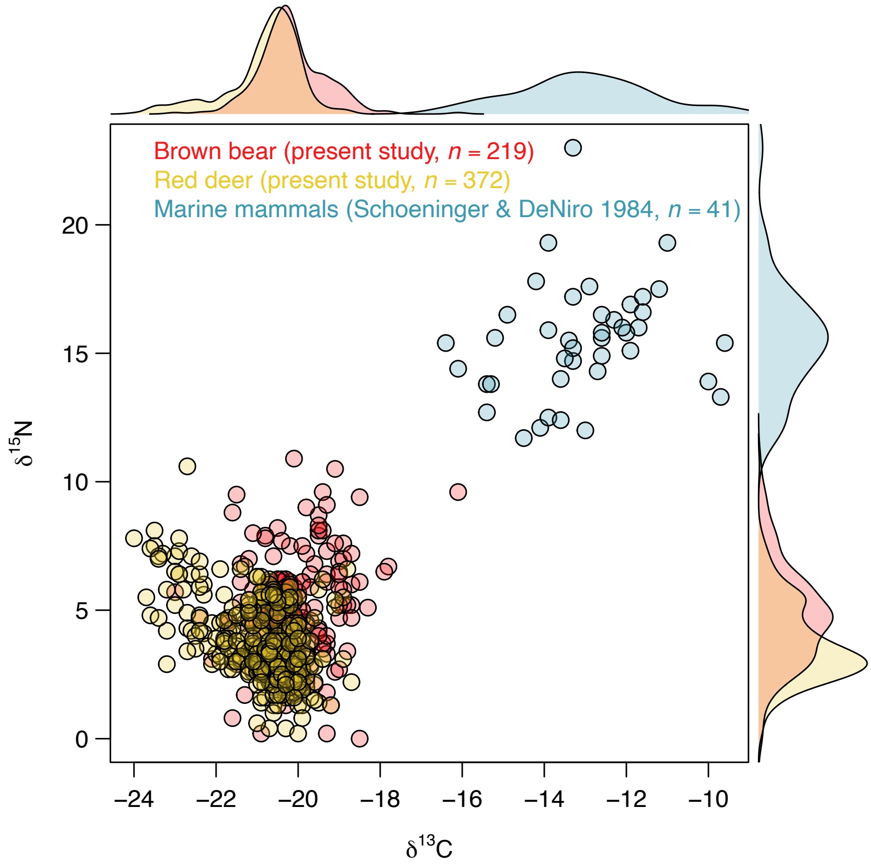

Supplementary Figure 3. Comparison of stable isotope ratios from the present study with marine mammals from the literature[8]. Shown are raw data (circles) and estimated probability density functions based on kernel density estimation. The distribution of samples from brown bear and red deer in the present study are very similar, but both strongly differ from the distribution of samples for a range of marine mammal species from the literature. Sample size is indicated by *n*.

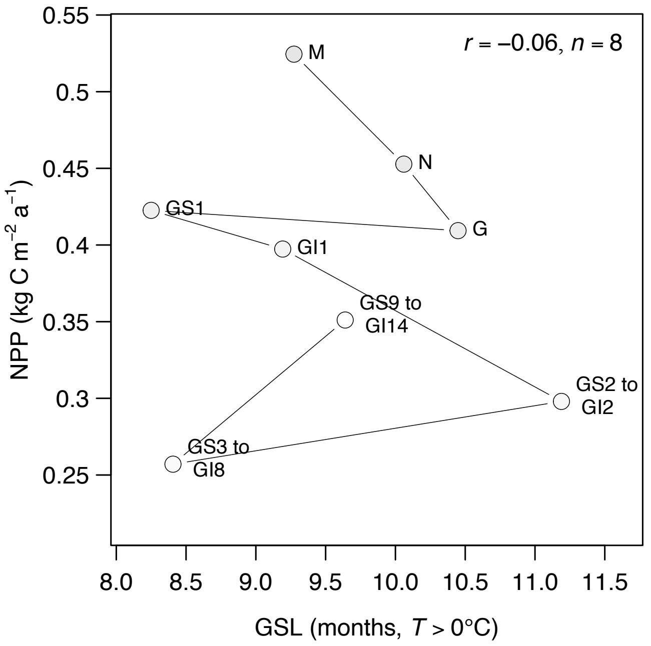

Supplementary Figure 4. Relationship between growing season length (GSL, months *T* > 0°C) and net primary productivity (NPP, kg C m^{-2} a^{-1}) across the eight time periods in the paleoecological data. Lines connect successive time periods. Abbreviations are GS9 to GI14, Greenland Stadial 9 to Greenland Interstadial 14; GS3 to GI8, Greenland Stadial 3 to Greenland Interstadial 8; GS2 to GI2, Greenland Stadial 2 to Greenland Interstadial 2; GI1, Greenland Interstadial 1; GS1, Greenland Stadial 1; M, Meghalayan; N, Northgrippian; G, Greenlandian. NPP and GSL were only very weakly correlated (Pearson's $r = -0.06$, $t = -0.14$, df = 6, $P = 0.89$). Sample size is indicated by *n* (i.e., the number of time periods).

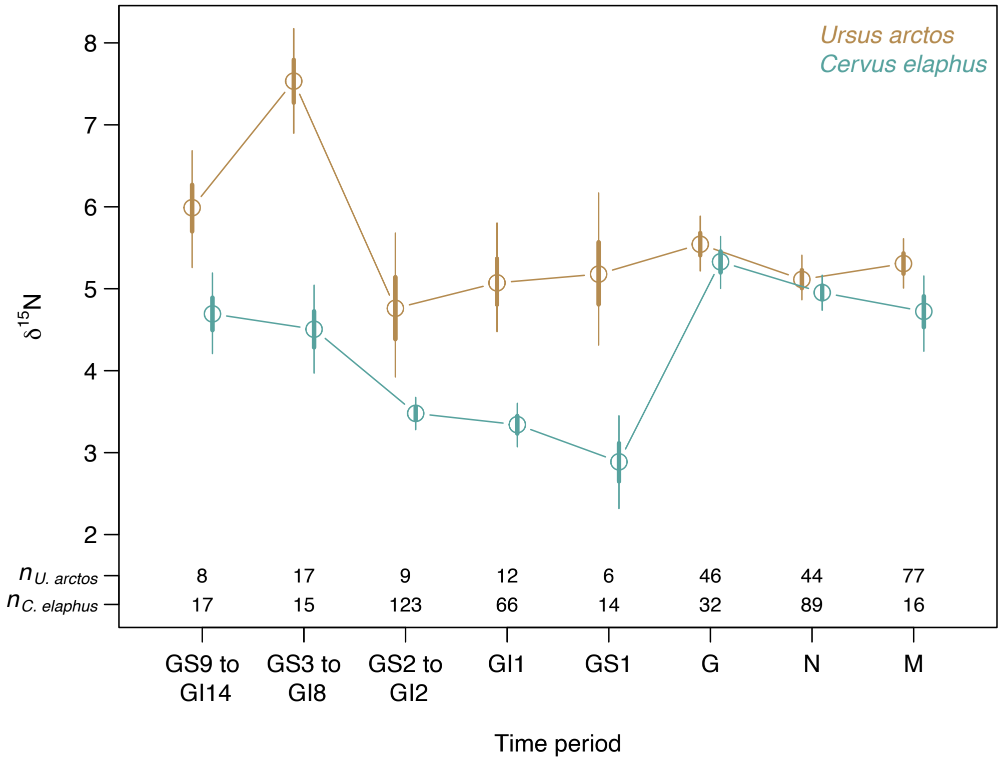

Supplementary Figure 5. Estimated bias-corrected δ^{15}N value of red deer and brown bear in each of the eight time periods. Median (circles) and associated 50%, and 90% Equal-Tailed Intervals (thick and thin lines) of the bias-corrected δ^{15}N values of red deer (purple) and brown bear (orange) based on sub-model I in the paleoecological model (Supplementary Table 3a). Abbreviations are GS9 to GI14, Greenland Stadial 9 to Greenland Interstadial 14; GS3 to GI8, Greenland Stadial 3 to Greenland Interstadial 8; GS2 to GI2, Greenland Stadial 2 to Greenland Interstadial 2; GI1, Greenland Interstadial 1; GS1, Greenland Stadial 1; M, Meghalayan; N, Northgrippian; G, Greenlandian. Sample sizes for brown bear ($n_{U.\ arctos}$) and red deer ($n_{C.\ elaphus}$) are given at the bottom of the plot.

References

1. Hewitt, D. G. & Robbins, C. T. Estimating Grizzly Bear Food Habits from Fecal Analysis. *Wildl. Soc. Bull.* **24**, 547–550 (1996).
2. Johansen, T. The diet of the brown bear (Ursus arctos) in central Sweden. (Norwegian University of Science and Technology, Trondheim, 1997).
3. Dahle, B., Sørensen, O., Wedul, E., Swenson, J. E. & Sandegren, F. The diet of brown bears Ursus arctos in central Scandinavia: effect of access to free-ranging domestic sheep Ovis aries. *Wildl. Biol.* **4**, 147–158 (1998).
4. Persson, I.-L., Wikan, S., Swenson, J. E. & Mysterud, I. The diet of the brown bear Ursus arctos in the Pasvik Valley, northeastern Norway. *Wildl. Biol.* **7**, 27–37 (2001).
5. Bojarska, K. & Selva, N. Correction factors for important brown bear foods in Europe. *Ursus* **24**, 13–15 (2013).
6. Pritchard, G. T. & Robbins, C. T. Digestive and metabolic efficiencies of grizzly and black bears. *Can. J. Zool.* **68**, 1645–1651 (1990).
7. Dormann, C. F. *et al.* Collinearity: a review of methods to deal with it and a simulation study evaluating their performance. *Ecography* **36**, 27–46 (2013).
8. Schoeninger, M. J. & DeNiro, M. J. Nitrogen and carbon isotopic composition of bone collagen from marine and terrestrial animals. *Geochim. Cosmochim. Acta* **48**, 625–639 (1984).
