# Supplementary material for: Dynamic omnivory shapes the functional role of large carnivores under global change
Source: Nat Commun. 2025 Dec 3;16:10896. doi: 10.1038/s41467-025-65959-7 (PMC12678555; doi:10.1038/s41467-025-65959-7)
Supplement: Supplementary file 2 — Reporting Summary [file 41467_2025_65959_MOESM2_ESM.pdf]

nature portfolio

Corresponding author(s): Jörg Albrecht

Last updated by author(s): Sep 8, 2025

Reporting Summary

Nature Portfolio wishes to improve the reproducibility of the work that we publish. This form provides structure for consistency and transparency in reporting. For further information on Nature Portfolio policies, see our Editorial Policies and the Editorial Policy Checklist.

Statistics

For all statistical analyses, confirm that the following items are present in the figure legend, table legend, main text, or Methods section.

n/a	Confirmed	
☐	☒	The exact sample size (*n*) for each experimental group/condition, given as a discrete number and unit of measurement
☐	☒	A statement on whether measurements were taken from distinct samples or whether the same sample was measured repeatedly
☐	☒	The statistical test(s) used AND whether they are one- or two-sided<br>*Only common tests should be described solely by name; describe more complex techniques in the Methods section.*
☐	☒	A description of all covariates tested
☐	☒	A description of any assumptions or corrections, such as tests of normality and adjustment for multiple comparisons
☐	☒	A full description of the statistical parameters including central tendency (e.g. means) or other basic estimates (e.g. regression coefficient) AND variation (e.g. standard deviation) or associated estimates of uncertainty (e.g. confidence intervals)
☐	☒	For null hypothesis testing, the test statistic (e.g. *F*, *t*, *r*) with confidence intervals, effect sizes, degrees of freedom and *P* value noted<br>*Give P values as exact values whenever suitable.*
☐	☒	For Bayesian analysis, information on the choice of priors and Markov chain Monte Carlo settings
☐	☒	For hierarchical and complex designs, identification of the appropriate level for tests and full reporting of outcomes
☐	☒	Estimates of effect sizes (e.g. Cohen's *d*, Pearson's *r*), indicating how they were calculated

Our web collection on statistics for biologists contains articles on many of the points above.

Software and code

Policy information about availability of computer code

Data collection	All data was originally stored as csv files (handled with Microsoft Excel version 16.86) and imported into R (version 4.4.2) using the R Studio (version 2024.12.0+467) programme.
Data analysis	All data analyses was conducted in R (version 4.4.2) and JAGS (version 4.3) via R Studio (version 2024.12.0+467) and the following R packages: coda (0.19-4.1), LaplacesDemon (16.1.6), rjags(4-16), Bchron (version 4.7.6), pastclim (version 2.2.0), terra (version 1.8-29), sf (version 1.0-19), sp (version 2.2-0), raster (version 3.6-31), and ape (version 5.8-1). The code used for the analysis and creating the figures is deposited together with the raw data in figshare with the identifier https://doi.org/10.6084/m9.figshare.25671048.

For manuscripts utilizing custom algorithms or software that are central to the research but not yet described in published literature, software must be made available to editors and reviewers. We strongly encourage code deposition in a community repository (e.g. GitHub). See the Nature Portfolio guidelines for submitting code & software for further information.

Data

Policy information about availability of data

All manuscripts must include a data availability statement. This statement should provide the following information, where applicable:

- Accession codes, unique identifiers, or web links for publicly available datasets
- A description of any restrictions on data availability
- For clinical datasets or third party data, please ensure that the statement adheres to our policy

The data generated in this study have been deposited in figshare with the identifier https://doi.org/10.6084/m9.figshare.25671048. The original specimens analyzed in this study are deposited in the National Historical Museums (Stockholm, Sweden), the Lund University Historical Museum (Lund, Sweden), the Nationalmuseum Jamtli (Östersund, Sweden), the Archaeological and Ethnographic Museum (Łódź, Poland), the Department of Environmental Archaeology and Human Paleoecology, Nicolaus Copernicus University (Toruń, Poland), the Institute of Archaeology, Nicolaus Copernicus University (Toruń, Poland), the Department of Paleoenvironmental Research, Adam Mickiewicz University (Poznań, Poland), the Department of Paleozoology at the University of Wrocław (Wrocław, Poland), the Museum of Archaeology, Wrocław City Museum (Wrocław, Poland), the Institute of Systematics and Evolution of Animals, Polish Academy of Sciences (Kraków, Poland), the National Museum (Prague, Czech Republic), the Institute of Archaeology of the Czech Academy of Sciences (Prague, Czech Republic), the Moravian Museum (Brno, Czech Republic), the Museum of Bohemian Karst (Beroun, Czech Republic). Detailed information on the deposition (institution and accession number) and provenance of each specimen (locality, country, and geographic coordinates) is provided in the raw data file available in the public repository (data/paleoData/paleoDatabase_20250903.xlsx).

Research involving human participants, their data, or biological material

Policy information about studies with human participants or human data. See also policy information about sex, gender (identity/presentation), and sexual orientation and race, ethnicity and racism.

Reporting on sex and gender	The research did not involve human participants.
Reporting on race, ethnicity, or other socially relevant groupings	The research did not involve human participants.
Population characteristics	The research did not involve human participants.
Recruitment	The research did not involve human participants.
Ethics oversight	The research did not involve human participants.

Note that full information on the approval of the study protocol must also be provided in the manuscript.

Field-specific reporting

Please select the one below that is the best fit for your research. If you are not sure, read the appropriate sections before making your selection.

☐ Life sciences ☐ Behavioural & social sciences ☒ Ecological, evolutionary & environmental sciences

For a reference copy of the document with all sections, see nature.com/documents/nr-reporting-summary-flat.pdf

Ecological, evolutionary & environmental sciences study design

All studies must disclose on these points even when the disclosure is negative.

Study description	We combined macroecological and paleoecological approaches to investigate how large terrestrial omnivores adapt their trophic position in food webs to changing net primary productivity and growing season length.
Research sample	We focus on the seven extant terrestrial bear species (Order: Carnivora, Family: Ursidae), which are the largest terrestrial omnivores and occupy a wide range of biomes from the arctic tundra to tropical rainforests. Unlike most other large carnivores, bears show a preference for low-protein diets and have relatively weak craniodental adaptations to carnivory, which allows them to maintain a high degree of dietary flexibility. Owing to their broad dietary niches, bears contribute to a multitude of ecosystem processes, such as predation, scavenging, or frugivory that can have strong impacts on prey populations, plant regeneration, nutrient cycling and energy fluxes within and across terrestrial and aquatic ecosystems.
Sampling strategy	We first used a macroecological approach to investigate how the trophic position of extant bears is related to NPP and growing season length across their geographic ranges. To do so, we compiled a comprehensive database of dietary compositions from the literature based on micro-histological analyses of fecal and stomach contents throughout the geographic ranges of the seven extant terrestrial bear species (n = 210 records from n = 155 studies).<br>In a second step, we used a paleoecological approach to investigate how European brown bears adapted their trophic position to the marked increases in NPP and growing season length at the transition from the Late Pleistocene to the Holocene. To do so, we

compiled a comprehensive database of stable isotope measurements based on fossil and subfossil bone and tooth remains of brown bears (n = 219) and red deer (Cervus elaphus, n = 372) across Europe, covering the last 55,000 years before present.

Data collection	Macroecological data: To obtain dietary data for the seven extant terrestrial bear species, we conducted a literature search in the Web of Science Core Collection for publications until 2018. We searched titles and abstracts using the keywords "(food OR feed* OR diet* OR forag* OR nutri* OR scat* OR fec* OR faec* OR stomach* OR gut*) AND (ursidae OR ursus OR melursus OR tremarctos OR helarctos OR ailuropoda OR bear* OR panda)". We also checked the reference lists of the retrieved publications for additional studies that had not been identified by the keyword search.<br>Paleoecological data: To reconstruct the trophic position of the European brown bear during the Late Pleistocene and Holocene, we compiled a database of 591 dated and georeferenced subfossil and fossil remains of brown bears and red deer using published sources (n = 483 samples) and additional material from museum collections (n = 108).
Timing and spatial scale	The macroecological analysis was based on a global collection of dietary studies on the seven extant terrestrial bear species that were published until 2018. The paleoecological analysis was focused on specimens of brown bear and red deer that covered a period from 55,000 years before present to the present. All museum specimens used in the paleoecological analysis were from excavations on the European continent.
Data exclusions	We excluded the polar bear (Ursus maritimus) from our analysis, because this species almost exclusively hunts for marine prey on Arctic Sea ice, but fasts on land during the ice-free season. Therefore, the minor contribution of terrestrial food sources during the ice-free season is not representative of the species' trophic niche. As the study focuses on terrestrial ecosystems we restricted the analyses to the remaining seven extant terrestrial bear species.
Reproducibility	All stable isotope measurements can in principle be repeated/replicated, as the original specimens are still available/stored in the museums where they have been collected. Furthermore, the extracted collagen, if not fully depleted in the course of analysis, is also available for further research (stored at the University of Tübingen). Since all raw data along with the code used for the analyses are published in a public repository, the statistical estimates of European brown bear diet during the Late Pleistocene and Holocene can be reproduced.
Randomization	Since the analysis was based on data from the literature and museum collections, randomization was not possible. However, the original studies from the primary literature collected the data to describe local bear species' diets, without considering broader climate or ecosystem productivity contexts. Therefore, we assume that the samples represent unbiased dietary descriptions that can be used to analyse the relationships of trophic position with net primary productivity and growing season length at macroecological and paleoecological scales.
Blinding	Not applicable to our study design/context as paleontological and archeological specimens had to be sampled based on preservation and reliability of taxonomic determination, leaving little possibilities for randomization or blinding.

Did the study involve field work? ☐ Yes ☒ No

Reporting for specific materials, systems and methods

We require information from authors about some types of materials, experimental systems and methods used in many studies. Here, indicate whether each material, system or method listed is relevant to your study. If you are not sure if a list item applies to your research, read the appropriate section before selecting a response.

Materials & experimental systems

n/a	Involved in the study	
☒	☐	Antibodies
☒	☐	Eukaryotic cell lines
☐	☒	Palaeontology and archaeology
☒	☐	Animals and other organisms
☒	☐	Clinical data
☒	☐	Dual use research of concern
☒	☐	Plants

Methods

n/a	Involved in the study	
☒	☐	ChIP-seq
☒	☐	Flow cytometry
☒	☐	MRI-based neuroimaging

Palaeontology and Archaeology

Specimen provenance	The provenance of each specimen (locality, country, and geographic coordinates) is provided in the raw data file available in the public repository (https://doi.org/10.6084/m9.figshare.25671048; data/paleoData/paleoDatabase_20250903.xlsx). The original specimens analyzed in this study are curated in the National Historical Museums (Stockholm, Sweden), the Lund University Historical Museum (Lund, Sweden), the Nationalmuseum Jamtli (Östersund, Sweden), the Archaeological and Ethnographic Museum (Łódź, Poland), the Department of Environmental Archaeology and Human Paleoecology, Nicolaus Copernicus University (Toruń, Poland), the Institute of Archaeology, Nicolaus Copernicus University (Toruń, Poland), the Department of Paleoenvironmental Research, Adam Mickiewicz University (Poznań, Poland), the Department of Paleozoology at the University of Wrocław (Wrocław, Poland), the Museum of Archaeology, Wrocław City Museum (Wrocław, Poland), the Institute of Systematics and Evolution of Animals, Polish Academy of Sciences (Kraków, Poland), the National Museum (Prague, Czech Republic), the Institute of Archaeology of the Czech

	Academy of Sciences (Prague, Czech Republic), the Moravian Museum (Brno, Czech Republic), and the Museum of Bohemian Karst (Beroun, Czech Republic). Permission to sample the specimens for stable isotope analysis and radiocarbon dating was formally granted by each of the respective institutions, in accordance with their ethical guidelines and collection management policies. All sampling was carried out under these approved protocols and in collaboration with local scientists and curators to ensure the preservation and integrity of the collections. Permits for collection and storage of samples were issued to the Institute of Nature Conservation of the Polish Academy of Sciences in Kraków by the General Directorate for Environmental Protection (Poland; permit number: DZP-WG.6401.08.4.2014.JRO,kka; date of issue: 04.06.2014).
Specimen deposition	The sampled material used for stable isotope analysis is stored at the Department of Geosciences, University of Tübingen (Tübingen, Germany). The original specimens are deposited in the National Historical Museums (Stockholm, Sweden), the Lund University Historical Museum (Lund, Sweden), the Nationalmuseum Jamtli (Östersund, Sweden), the Archaeological and Ethnographic Museum (Łódź, Poland), the Department of Environmental Archaeology and Human Paleoecology, Nicolaus Copernicus University (Toruń, Poland), the Institute of Archaeology, Nicolaus Copernicus University (Toruń, Poland), the Department of Paleoenvironmental Research, Adam Mickiewicz University (Poznań, Poland), the Department of Paleozoology at the University of Wrocław (Wrocław, Poland), the Museum of Archaeology, Wrocław City Museum (Wrocław, Poland), the Institute of Systematics and Evolution of Animals, Polish Academy of Sciences (Kraków, Poland), the National Museum (Prague, Czech Republic), the Institute of Archaeology of the Czech Academy of Sciences (Prague, Czech Republic), the Moravian Museum (Brno, Czech Republic), and the Museum of Bohemian Karst (Beroun, Czech Republic). Detailed information on the deposition of each specimen (institution and accession number) is provided in the raw data file available in the public repository (https://doi.org/10.6084/m9.figshare.25671048; data/paleoData/paleoDatabase_20250903.xlsx).
Dating methods	Dates of specimens for which stable isotope analysis has been conducted in the present study were based on direct radiocarbon (14C) dating (n = 55) or based on the context of the excavation sites (n = 53). Contextual dates were obtained based on related artifacts. Radiocarbon dating was done using accelerator mass spectrometry at the Poznań Radiocarbon Laboratory (Poz), Poznań, Poland.

☒ Tick this box to confirm that the raw and calibrated dates are available in the paper or in Supplementary Information.

Ethics oversight	The original specimens analyzed in this study are curated in the National Historical Museums (Stockholm, Sweden), the Lund University Historical Museum (Lund, Sweden), the Nationalmuseum Jamtli (Östersund, Sweden), the Archaeological and Ethnographic Museum (Łódź, Poland), the Department of Environmental Archaeology and Human Paleoecology, Nicolaus Copernicus University (Toruń, Poland), the Institute of Archaeology, Nicolaus Copernicus University (Toruń, Poland), the Department of Paleoenvironmental Research, Adam Mickiewicz University (Poznań, Poland), the Department of Paleozoology at the University of Wrocław (Wrocław, Poland), the Museum of Archaeology, Wrocław City Museum (Wrocław, Poland), the Institute of Systematics and Evolution of Animals, Polish Academy of Sciences (Kraków, Poland), the National Museum (Prague, Czech Republic), the Institute of Archaeology of the Czech Academy of Sciences (Prague, Czech Republic), the Moravian Museum (Brno, Czech Republic), and the Museum of Bohemian Karst (Beroun, Czech Republic). Permission to sample the specimens for stable isotope analysis and radiocarbon dating was formally granted by each of the respective institutions, in accordance with their ethical guidelines and collection management policies. All sampling was carried out under these approved protocols and in collaboration with local scientists and curators to ensure the preservation and integrity of the collections. Curators of the above institutes were actively involved in evaluating and approving the study design and contributed to discussions regarding both the scope of the research and the interpretation of the results.

Note that full information on the approval of the study protocol must also be provided in the manuscript.

Plants

Seed stocks	not applicable
Novel plant genotypes	not applicable
Authentication	not applicable
